# The Impact of a Pandemic COVID-19 on the Incidence of Borreliosis in Poland

**DOI:** 10.1007/s11686-021-00495-0

**Published:** 2022-01-03

**Authors:** Mariusz Piotrowski, Anna Rymaszewska

**Affiliations:** Institute of Biology, University of Szczecin, 70-453 Szczecin, Poland

**Keywords:** Borreliosis, Lyme disease, Poland, COVID-19, SARS-CoV-2

## Abstract

**Purpose:**

Lyme disease is the most common tick-borne disease, caused by spirochetes of the genus *Borrelia*, transmitted by ticks of the *Ixodes* genus in Poland. The purpose of this analysis was whether the COVID-19 outbreak had a significant impact on the number of reported Lyme disease cases.

**Materials and Methods:**

The data included in the World Health Organization (WHO) and the data from the “Reports on incidence of infectious diseases, infections and poisoning in Poland” presented by the Department of Epidemiology NIZP-PZH were analyzed.

**Results:**

To the end of 2020, there were registered 12, 524 Lyme disease cases. In the same period, in 2018 and 2019 were registered, respectively, 20, 150 and 20, 614 Lyme disease cases. The overall number of Lyme disease cases in 2018 and 2019 was at a similar level. The monthly increase in the number of cases was also at a similar level. The year 2020 in January and February was characterized by the same increase in the number of cases as in previous years. The difference started to be noticeable in March and the lowered growth compared to the previous years has been maintained to this day. In December, about 8, 000 fewer cases of Lyme disease were registered than in previous years.

**Conclusion:**

The reduced number of cases of Lyme disease coincided with the beginning of the COVID-19 epidemic in Poland in March 2020. Every year, the incidence of Lyme disease in Poland is at a similar level with a similar monthly increase. The outbreak of the COVID-19 pandemic had a significant impact on the number of cases recorded, which could have catastrophic consequences for people who did not receive treatment in the right time.

## Introduction

The novel coronavirus disease (COVID-19), induced by severe acute respiratory syndrome coronavirus 2 (SARS-CoV-2) and first reported in late December 2019 in Wuhan, China, quickly became an emerging, rapidly evolving situation, spreading inevitably outside China and the Asian continent, and it was declared a pandemic in March 2020 [1], 2. Under these circumstances, different countries confirming their first cases began to implement a strict hygiene regime and eventually imposed city-wide and national lockdown measures. As a result, an estimated 4 billion people were forced to quarantine themselves at home. According to data from the World Health Organization (WHO), the coronavirus disease (COVID-19) has spread to almost all countries around the world. As of 10 January 2021, the number of confirmed cases was 88,383,771 and the number of deaths was 1,919,126 [6].

The current goal of governments and ministries of health is to implement urgent measures to minimize the number of people infected with SARS-CoV-2. While it is of utmost importance to focus on controlling this communicable disease, a pandemic could also have long-term effects on people with non-communicable diseases.

Although there is no peer-reviewed scientific evidence on this issue yet, initial reports from scientific societies in Italy and Spain suggest a substantial reduction in admission for stroke or Myocardial infarction since the start of the outbreak, for example. In Spain, cardiologists have seen a 40% reduction in heart attack treatments and a decrease in the number of diagnostic procedures. One hypothesis is that individuals do not go to the hospital even if they need to. There is also a shortage of healthcare staff to cover both SARS-CoV-2-related illness and all other routine medical care [3].

In Poland, one of the non-communicable diseases that affects 20,000 people every year is Lyme disease, which is a mandatorily notifiable disease entity, therefore precise data are available [4]. Lyme disease, known also as borreliosis is caused by spirochaetes belonging to the *Borrelia burgdorferi* sensu lato complex, carried by *Ixodes* ticks. *B. burgdorferi* is one of the most significant human pathogens transmitted by ticks [5].

## Epidemiological Situation of Lyme Disease in Poland After the Start of the COVID-19 Epidemic in 2020

The epidemiological situation of Lyme disease in Poland based on the data from the “Reports on incidence of infectious diseases, infections and poisoning in Poland” presented by the Department of Epidemiology NIZP-PZH.

To the end of 2020, there were registered 12,524 Lyme disease cases. In the same period, in 2018 and 2019, were registered, respectively, 20,150 and 20,614 Lyme disease cases. Incidence rate in 2020 in Poland was 32,63 per 100,000 population and it was much lower, i.e., by 19,83 and 21,03 in comparison to the incidence rate in 2018 and 2019, respectively (Table 1).

**Table 1:** Increase in the incidence of Lyme disease in Poland in 2018, 2019 and 2020. Number of cases and incidence per 100,000 population

	2018		2019		2020	
	No of cases	Incidence/100,000	No of cases	Incidence/100,000	No of cases	Incidence/100,000
January	1118	2,91	916	2,39	913	2,38
February	2172	5,65	1934	5,04	1934	5,04
March	3261	8,49	2954	7,70	2257	5,88
April	4232	11,02	3809	9,92	2509	6,54
May	5485	14,28	4964	12,93	2985	7,78
June	7349	19,13	6685	17,41	4259	11,10
July	9757	25,40	9333	24,31	6435	16,76
August	12586	32,76	11876	30,94	7847	20,44
September	14762	38,43	14288	37,22	9376	24,43
October	17049	44,38	16788	43,70	9857	25,68
November	18753	48,82	19020	49,51	10710	27,90
December	20150	52,46	20614	53,66	12524	32,63

**Fig. 1:**
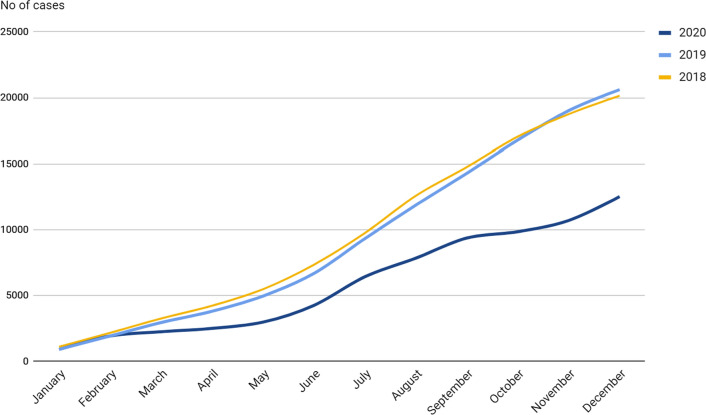
Monthly increase in the incidence of Lyme disease in 2018, 2019 and 2020

The overall number of Lyme disease cases in 2018 and 2019 was at a similar level. The monthly increase in the number of cases was also at a similar level. The year 2020 in January and February was characterized by the same increase in the number of cases as in previous years. The difference started to be noticeable in March and the lowered growth compared to the previous years has been maintained to this day. In December, about 8 000 fewer cases of Lyme disease were registered than in previous years. The reduced number of cases of Lyme disease coincided with the beginning of the COVID-19 epidemic in Poland in March 2020 (Fig. 1).

## Summary and conclusion

The first case of COVID-19 in Poland was confirmed on 4 March 2020. School and university closure was imposed on 11 March; on 15 March, borders were closed to foreigners; from 24 March, a nationwide lockdown was imposed; on 20 April, the ban on the recreational use of forests and parks was lifted; on 4 May, hotels and shopping centers were permitted to reopen; and since 6 May, daycare centers and kindergartens have been allowed to welcome their pupils again. In other words, for the majority of Poles, the stay-at-home order encompassed six weeks. The on-going pandemic (COVID-19), caused by the coronavirus SARS-CoV-2, has dramatically impacted on people from public health. One consequence of quarantine measures was a decrease in physical activity levels in many individuals. In addition to laws limiting access to outdoor space. During the SARS-CoV-2 outbreak, healthcare systems began postponing and scaling down some aspects of routine management, outpatient visits, and non-urgent surgery to avoid unnecessary hospital visits, reduce the burden on hospitals, and decrease infection risk. Although there are no data available yet on this issue, it is likely that many patients have decreased access to outpatient visits and one-on-one clinical advice, and in some cases, there may be a shortage of medicines. Further, some patients may be reluctant to seek care due to fears of infection in healthcare settings. The situation is further exacerbated by the preexisting European shortage of skilled healthcare workers and that many healthcare workers have been infected with SARS-CoV-2, which affects staffing levels.

The above situation is very well illustrated in the example of Lyme disease in Poland. Every year, the incidence of Lyme disease in Poland is at a similar level with a similar monthly increase. The outbreak of the COVID-19 pandemic had a significant impact on the number of cases recorded, which could have catastrophic consequences for people who did not receive treatment in the right time.
